# Supplementary material for: The concept of optimal planning of a linearly oriented segment of the 5G network
Source: PLoS One. 2024 Apr 17;19(4):e0299000. doi: 10.1371/journal.pone.0299000 (PMC11023492; doi:10.1371/journal.pone.0299000)
Supplement: S1 File — (DOCX) [file pone.0299000.s001.docx]

**Nomenclature**

$B=\left\{b_{i}\right\}$, $i=\overline{1,n}$, is a set of multiband base stations, where $b_{i}=\left\{s_{i},\left\{\vec{S}_{ij}\right\},t_{i},v_{i}\right\}$, $j=\overline{1,n}$, $i\neq j$;

$s_{i}$ is the coverage semidiameter of the service supported by the base station $b_{i}$, focused on users' tasks maintenance;

$\left\{\vec{S}_{ij}\right\}$ is the coverage semidiameter of the service supported by the base station $b_{i}$, focused on the connection of the $і$-th base station with the $j$-th base station, $b_{i},b_{j}\in B$;

$t_{i}$ is the bandwidth of the base station $b_{i}$;

$v_{i}$ is a cost of the base station $b_{i}$;

$V$ is the maximum amount of funds that can be spent on the deployment of the network architecture $В$;

$D$ is the maximum permissible delay in the transmission of a data packet between the users of the network $В$;

$\rho=\left[p_{0},p_{m+1}\right]$ is a linear segment on which the network architecture $В$ is deployed;

$l$ is the length of the linear segment $\rho$;

$p_{0}$ and $р_{m+1}$ are the starting and ending points of the segment $\rho$, respectively;

$Р=\left\{p_{k}\right\}$ is a set of points that are admissible for placing base stations of the network $В$ within the segment $\rho$;

$x_{k}$ is a coordinate of the point $p_{k}$ within the segment $\rho$, $\forall k$;

$A=\left\{\left(p_{k},b_{i}\right)\right\}$ is an acceptable placement plan of base stations from the set $В$, $p_{k}\in P$, $k\in\left\{\overline{1,m}\right\}$;

$h\left(A\right)=l-y\left(A\right)$ is a the of non-coverage of a segment $\rho$;

$W$ is a set of all admissible placement plans $А$;

$\Omega$ is a set of options for placing base stations from the set $В$ on the given set $Р$;

$W_{с}$ is a redefinition of the set $\Omega$, where the subscript $с$ fixes the iteration number;

$\beta_{ki}$ is a rule that determines the division (2), (3);

$B=\left\{\beta_{ki}\right\}$ is a set of partitioning rules;

$B^{+}\in B$ is a subset, which includes all cases when $\beta_{ki}=1$;

$B^{-}\in B$ is a subset, which includes all cases when $\beta_{ki}=0$;

$B^{\sim}\in B$ is a subset, which includes all cases that are not affected by the value of $\beta_{ki}$ at the current $с$-th iteration;

$\hat{А}$ is the best version of the placement plan $\hat{А}$ among those already found;

$z$ is the index of the base station from the set $В$ located at the point closest to the left $p_{d}$;

$\gamma$ is the partial non-coverage function, which is defined for any two points $p_{q}$ and $p_{d}$ from the set $Р$ at $q>d$, in which the base stations $b_{t}$ and $b_{g}$ are located;

$R\left(W_{c}\right)\leq h\left(A\right)$ is the lower bound of the non-coverage function $h\left(A\right)$ for an arbitrary placement plan $А$, which corresponds to the set $W_{c}$;

$r_{1}\left(W_{c}\right)$ is a term of (6) calculated as the sum of the values of functions (4) to the left of the point $p_{q}$ and the value of the coverage semidiameter of the base station $b_{g}$ located at this point;

$r_{2}\left(W_{c}\right)$ is a term of (6) calculated as the sum of the values of functions (4) to the right of the point $p_{q}$, that is, on some fragment $\pi$ to the end of the segment $\rho$;

$u_{i}$ is a Boolean variable used in (7);

$\left|\pi\right|$ is the length of the fragment $\pi$;

$n$ is the number of unoccupied points for placing base stations from the set $В$ on the fragment $\pi$;

$\varepsilon$ is the percentage deviation for (11) necessary to calculate $\Delta=l\varepsilon>0$;

$D^{p2p}$ is a transfer delay between network endpoints (gateways);

$P_{tran}$ is the transmitter power;

$L_{tran}$, $L_{rec}$ are signal losses on the transmitter and receiver sides, respectively;

$G_{tran}$, $G_{rec}$ are the main characteristics of the transmitter and the receiver, respectively;

$L_{link}$ is a signal loss in the communication channel (air);

$S_{rec}$ is the sensitivity of the receiver;

$SFM$ is the Signal Fading Margin;

$F_{5G}$ is the central frequency of the 5G communication system;

$S_{t-r}$ is the distance between the transmitter and the receiver;

$K\left(F,S\right)$ is a constant, the value of which depends on the units of measurement of the characteristics $F$ and $S$;

$\eta_{i}=0.5t_{i}/r$ is the service intensity for the $b_{i}$-th base station;

$t_{i}$ is the throughput of the $b_{i}$-th base station;

$r$ is the average length of the data packet;

$n_{is}$ is the number of incoming flows;

$L_{t/r}=L_{tran}=L_{rec}$ characterizes signal loss; parameter $t$ characterizes a throughput of the base station;

$v$ is a parameter that characterizes the cost of placing base station $b_{i}\in B$ at point $p_{k}\in P$ with coordinates $х_{k}\in X$, $i=\overline{1,\left|B\right|}$, $k=\overline{1,\left|P\right|}$;

$\left\langle h,V,D,N\right\rangle$ is a metrics of qualitative indicators $h\left(\hat{A}\right)$, $V\left(\hat{A}\right)$, $D\left(\hat{A}\right)$, $N\left(\hat{A}\right)$, respectively (look at the Fig. 5);

$N\left(\hat{A}\right)$ is the number of the vertex of the search tree;

$\left\langle T,h,Acc\right\rangle$ is a metric of qualitative indicators such as the solution time $T$ [s], non-coverage $h$ [m] and solution accuracy $Acc$ [%], for the three options for calculating the term $r_{2}\left(W_{c}\right)$ from expression (6) described in Section 2.3 (look at Figs. 8-10).
