# Supplementary material for: The concept of optimal planning of a linearly oriented segment of the 5G network
Source: PLoS One. 2024 Apr 17;19(4):e0299000. doi: 10.1371/journal.pone.0299000 (PMC11023492; doi:10.1371/journal.pone.0299000)
Supplement: Attachment — Submitted filename: Responses.pdf [file pone.0299000.s004.pdf]

Response to the Reviewer #1 comments

Dear Colleague,

I thank you for supporting the publication of our article.

Also, I take this opportunity to congratulate you on the upcoming 2024 and wish you good health, prosperity and creative success.

Sincerely,
Viacheslav Kovtun,
corresponding author.

Response to the Reviewer #2 comments

Dear Colleague,

I thank you for the time you devoted to the article, as well as for the excellent advice, which significantly simplified the search for analogues of our research, and also made Section 1 more meaningful.

I have updated the article following your suggestions. At the same time, I tried to preserve the style of presentation of the material inherent in the scientific school to which I have the honour of belonging. I ask you to treat this with understanding. We all stand on the shoulders of giants.

I hope that you will perceive this version of the article positively and support its publication. And, of course, I congratulate you on the upcoming 2024 and wish you good health, prosperity and creative success.

Sincerely,
Viacheslav Kovtun,
corresponding author.

0. An optimal wireless network planning model requires evidence of input and innovation on geo-referenced scenarios. Innovation is not evident in recent publications, as expressed in https://ieeexplore.ieee.org/abstract/document/10233338, https://www.mdpi.com/1996-1073/13/1/97.

Dear Colleague, in response to this suggestion, we have added to Section 1 below the comparison of the studies mentioned by you in comparison with the mathematical apparatus proposed in our article.

The papers [30, 31] discuss the strategic planning and implementation of wireless heterogeneous networks for smart metering, employing a cross-layer approach. Our methodology integrates considerations from the network layer, addressing routing and flow demands at each link within the network, while simultaneously accounting for limitations at the physical layer, specifically the capacity constraints of short-range technology in a multi-hop configuration. The proposed model utilizes a column generation approach to address the capacitated multicommodity flow problem, incorporating wireless link capacities, coverage parameters, and associated costs. Those studies incorporate multi-hop routing of packets within a mesh network composed of smart meters and concentrators connected to a cellular network through base stations. Each link's traffic is represented in a multigraph with occupation percentages, and a scalable routing tree is constructed on a georeferenced map to simulate a real-world deployment. The outcomes illustrate the performance of the proposed model concerning concentrator traffic load, network coverage, and energy consumption reduction. The findings underscore that the integration of multi-hop short-range technology leads to a cost-effective infrastructure by diminishing the number of smart meters requiring a direct connection to cellular technology.

Unlike the previously mentioned studies, our research focuses on a specific optimization problem formulated in the context of organizing a wireless network

based on the 5G platform. The specificity is that the mentioned network is deployed along a linear segment, for example, a road, pipeline, or railway. A special feature of the 5G platform is, among other things, the ability to implement several virtual network segments on the base station hardware using Network Slicing technology. In the model proposed in Section 2.1, it is assumed that the frequency resource of the base stations (the cost of which is known) is divided into two virtual segments, one of which is used to serve subscribers, and the second to ensure the network connectivity (for communication with other base stations). The first segment is characterized by higher throughput and a smaller coverage radius, and the second – vice versa. For the deployed network, parameters such as total cost and total delay are taken into account. Within the target linear segment, the coordinates of the points at which the base station can be located are known. The special same type of base stations (gateways) are located at the ends of the target segment. The optimization problem is that it is necessary to place base stations on the target segment in such a way as to maximize the size of the telecommunications coverage of the segment while fulfilling the requirement for each station to communicate with gateways through a system of hosted stations, as well as fulfilling restrictions on the amount of end-to-end delay and the total cost of deployed stations. Thus, the wireless network structure obtained as a result of solving such an optimization problem will be reliable (due to constant communication between base stations and gateways) and efficient (due to maximizing coverage focused on serving subscribers). In addition, the gateway concept allows the integration of multiple line segments into a single network. This allows us to limit ourselves to the analysis of dozens of points of potential location of base stations in the process of searching for the optimal network configuration. In turn, this makes it possible to solve such an optimization problem using exact methods, among which the most suitable is the branch and bound method (subject to its adaptation to the above-formulated specifics of setting the optimization problem). In addition, the proposed mathematical apparatus takes into account that base stations (not gateways) can be of different types (this allows us to take into account the possibility of upgrading already functioning network architectures). Naturally, the last clarification further complicates the final optimization problem. All of the above stated in the complex is a new research problem, as a result of which it is possible to design linearly organized network segments based on 5G base stations that meet the conditions of high reliability, high quality of service and rational use of funds.

1.- Introduction (Generalities of the problem - 15 references between 2024-2020),
2.- Related Works (Specific works with punctual solutions, summary table of state of the art of other proposals against the current work - 15 references between 2024-2020),

Dear Colleague, I diligently tried to follow this suggestion to the letter, but I could not. First of all, I have not found any direct analogues for solving the optimization problem, formulated in terms set out in p. 0. The approximate analogues I have found (sources [11]-[31]) are somewhat beyond the lower limit you defined.

An indirect confirmation of the objectivity of my statement is that 40% of the references in the relevant and high-quality study *https://ieeexplore.ieee.org/abstract/document/10233338* you mentioned were published before 2020.

A summary of the metrics used in the analyzed analogues is presented in the form of the following text at the end of section 1:

Characteristic of the mentioned studies is the variety of methods used to solve the problems of optimal design of wireless network architectures, which does not eliminate the general drawback: the authors deliberately narrow the range of characteristics of the deployed base stations taken into account (most often, to two parameters – coverage semidiameter and cost). The authors also consider the characteristics of the placed base stations to be unified, ignoring that the actual values of the corresponding characteristics depend on the location of the device. At the same time, the aspect of network connectivity (the continuity of interaction between located base stations within the SON) is not considered. The motive of the authors is clear – they strive to move from multi-parameter optimization problems to single-parameter ones. This makes it possible to increase the probability of the existence of a feasible solutions region and simplifies the solution process as such, but makes the resulting models extremely abstracted from the real object.

Note that the final stage of designing a wireless network segment is estimating its performance characteristics to verify the compliance of the values of these assessments with the specified requirements [9, 10]. This estimation most often uses simulation modelling, which is a computationally intensive process. If the network characteristics do not meet the specified requirements, we have to return to the previous stages and repeat the iterative design process with new input data to obtain a new architecture for the wireless network segment.

3.- Problem Formulation and Methodology (Table of variables of the mathematical model, mathematical model, pseudocode, methodology flowchart, Big Oh Notation of the algorithm - Forecasting)

Dear Colleague, thank you for this comment. Indeed, Section 2 is full of many entered parameters, which are quite difficult to remember as you read the article. In response to this suggestion, I introduced a Nomenclature section before References. In addition, the material in Section 2.2 is supplemented with visualization of the Branching of a binary search tree and the Moving through the search tree processes (Fig. 1 and Fig. 2, respectively).

The Materials and Methods section is devoted to a consistent, mathematically correct presentation of the proposed methods in the context of the general model outlined in section 2.1. A graphical interpretation of this material would be cumbersome and imprecise. That is why the material is presented analytically with textual additions.

If you find any errors in the proposed mathematical apparatus, please let me know. I will gratefully analyze and respond to your substantive comments. I also note that the “Forecasting” problem is not statemented or solved in the article, and the issue of the computational complexity of the proposed methods is studied in section 4.

4. - Analysis of Results (Metrics performed in Matlab in PDF format or directly from Overleaf).

Dear Colleague, I do not use Matlab because this software package does not allow you to create independent applications, which can then be patented and used in practice.

Following the link:

https://drive.google.com/file/d/1M13v2U8sQj70rgAqJvBtdnKprX5LY_09/view?usp=sharing

I posted a project written in Python, which is the embodiment of the mathematical apparatus presented in the article. You can use this code as you wish. The only thing is that I ask you not to use it personally or pass it on to third parties. This code is my intellectual property.

The structure of the created software is shown in the figure below

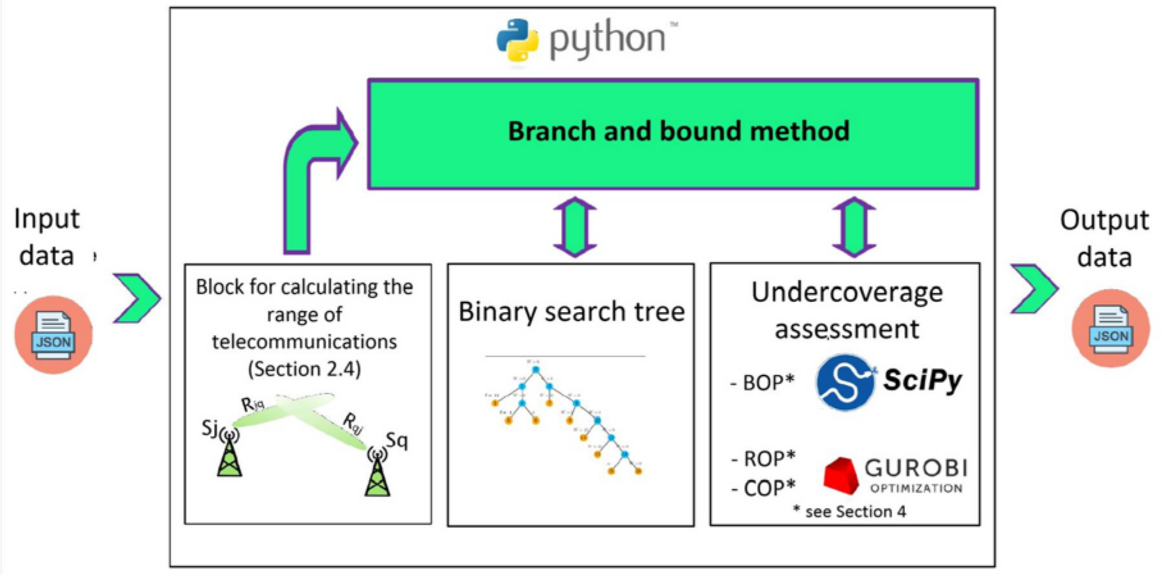

The input to the software package is a JSON file containing Configuration* parameters and Task input data**.

Before starting the placement task, it is necessary to prepare parameters for each base station (BS): communication radius between stations and BS coverage radius (Section 2.4). The parameters are calculated in the telecommunications range calculation block using the communication line energy potential equation and signal propagation models.

The base station placement problem is presented as a combinatorial model in extreme form. The algorithm for finding the optimal placement is of the branch and bound type. To find the optimal placement, a procedure for constructing a binary search tree is used. At each step, conditions are checked for the possibility of closing a tree node. The construction of a binary search tree is implemented recurrently.

As we move through the search tree, all received Optimal records are recorded (example in the figure below). The placement cost Cost, the end-to-end delay time Delay and the number of nodes in the tree (Node) where the record was obtained are also recorded. For each record, multiple station placements are recorded. The

last record obtained is the optimal solution to the problem. The solution to the problem is written in the output JSON file:

```
Optimal = 84.0, Cost = 11900,  Delay = 0.00107,  Node = 7
['S1', 'S2', 'S4', '-', '-', '-']
Optimal = 73.0, Cost = 11900,  Delay = 0.00107,  Node = 25
['S1', 'S2', '-', 'S4', '-', '-']
Optimal = 65.0, Cost = 12000,  Delay = 0.00107,  Node = 61
['S1', 'S3', '-', 'S5', '-', '-']
Optimal = 60.0, Cost = 11900,  Delay = 0.00107,  Node = 91
['S1', 'S4', '-', 'S2', '-', '-']
Optimal = 56.0, Cost = 12000,  Delay = 0.00107,  Node = 127
['S1', 'S5', '-', 'S3', '-', '-']
Optimal = 17.0, Cost = 11900,  Delay = 0.00107,  Node = 161
['S1', '-', 'S2', '-', 'S4', '-']
Optimal = 9.0, Cost = 11900,  Delay = 0.00107,  Node = 171
['S1', '-', 'S2', '-', '-', 'S4']
Optimal = 4.0, Cost = 12000,  Delay = 0.00107,  Node = 193
['S1', '-', 'S3', '-', '-', 'S5']
Optimal = 0.0, Cost = 12000,  Delay = 0.00107,  Node = 213
['S1', '-', 'S5', '-', '-', 'S3']
Total number of nodes is 298
```

* Configuration parameters are located under the configuration key:

```
"configuration": {
  "method": "bab",
  "estimation_method": "knapsack",
  "place_all_station": false,
  "drawing": false,
  "last_optimal_noncoverage": 0,
  "relative_deviation": null
},
```

– method: a method for solving the placement problem. The value bab is the branch and bound method or bf is the brute force method;
– estimation_method: a method for calculating the right under coverage estimate. The BOP value is a solution using the simplex method, the ROP value is a solution using an integer linear programming problem, or the COP value is a solution using the “Knapsack” problem (see Verification (7) in Section 2.3);
– placed_all_station: a special case of the placement problem, when there are no restrictions on cost and delay time, in such a setting the entire set of stations is placed. Accepts the values True or False;
– drawing: visualization of the resulting decision tree. Accepts the values True or False;
– last_optimal_noncoverage: the obtained optimal undercover on the last solution for the problem of finding a sequence of best solutions. Accepts any positive real values;
– relation_deviation: specified deviation in percentage from the obtained optimal solution for the problem of finding a sequence of best solutions.
** The JSON file contains the optimization Task input data:
– placement coordinates and gateway_placement coordinates;
– parameters of antennas of gateways and user_device subscriber devices;
– task restrictions on cost cost_limit and delay time delay_limit;
– parameters for calculating the range of telecommunication coverage: carrier frequency frequency, signal fading margin link_som and coverage_som;

– parameters for calculating delays: average packet size average_packet_size and intensity of incoming flow arrival_rate;
– sta – set of specified base stations.
Each element of the sta array is a JSON object and contains the following parameters:


```json
"sta": [
  {
    "Ptr_link": 19,
    "Gtr_link": 2,
    "Precv_link": -77,
    "L_link": 1,
    "L_coverage": 1,
    "Precv_coverage": -67,
    "Grecv_coverage": 5,
    "cost": 4600,
    "throughput": 72.2
  }
]
```


– Ptr_link – the power of the antenna providing telecommunications communication between stations;
– Gtr_link – antenna gain that provides telecommunications between stations;
– Precv_link – sensitivity of the antenna providing telecommunications communication between stations;
– L_link – signal loss in the cable and connectors of the transmitting path of the antenna, which provides telecommunications between stations;
– L_coverage – signal loss in the cable and connectors of the transmitting path of the antenna providing telecommunications coverage of the BS;
– Precv_coverage – sensitivity of the antenna providing telecommunications coverage of the BS;
– Grecv_coverage – a gain of the antenna providing telecommunications coverage of the BS;
– cost – the cost of the BS;
– throughput – BS throughput.

5.- Conclusions (Direct relation between the objective stated in the abstract vs. the metrics found)

Dear Colleague, a complete and detailed study of the proposed mathematical apparatus in the mathematical apparatus mentioned above (in the software description) is presented in section 4 (Discussion). This was done deliberately because this is a large work, the presentation of which in the conclusions section, firstly, will make the latter redundant, and secondly, the conclusions relate to the entire work as a whole, and not just its experimental part.

6.-References 2024-2020 (All documents with DOI from ScienceDirect, MDPI, Wiley, PLOS, Taylor & Francis, Springer, Hindawi, IEEE Xplore [transactions, magazines, journals]).

Done, dear Colleague (subject to the exceptions highlighted by me during my answer to suggestions 0, and 1).
