# Supplementary material for: The concept of optimal planning of a linearly oriented segment of the 5G network
Source: PLoS One. 2024 Apr 17;19(4):e0299000. doi: 10.1371/journal.pone.0299000 (PMC11023492; doi:10.1371/journal.pone.0299000)
Supplement: Attachment — Submitted filename: Responses.doc [file pone.0299000.s003.doc]

**Response to the Reviewer #1 comments**

Dear Colleague,

I am sincerely grateful to you for the time you devoted to the article and the positive reception of this research. I carefully revised the text of the article following your recommendations. I hope you will support the current version of the article.

With respect,

on behalf of the team of authors,

Viacheslav Kovtun.

*1. Provide the most important numerical experimental results at the end of the abstract.*

*2. Provide paper structure at the end of the abstract.*

Dear Colleague, in response to your remarks, I added the following information at the end of abstract:

The article presents a numerical example of solving an instance of the corresponding extremal problem. It is shown that the presented mathematical apparatus allows for the formation of a set of optimal placements taking into account the size of the non-coverage of the target area. To calculate this characteristic parameter, both exact and two approximate approaches are formalized. The results of the experiment showed that for high-dimensional problems, the approximate approach allows for reducing the computational complexity of implementing the adapted branch-and-bounds method by more than six times, with a slight loss of accuracy of the optimal solution. The structure of the article includes Section 1 (introduction and state-of-the-art), Section 2 (statement of the research, proposed models and methods devoted to the research topic), Section 3 (numerical experiment and analysis of results), and Section 4 (conclusions and further research).

*3. Literature survey should be expanded with practical and successful solutions to 5G optimization problems.*

In response to this remark, dear Colleague, the following fragment of text was added to Section 1:

The challenge of determining the optimal locations for 5G base stations can be conceptualized as a network design problem involving relays (NDPR), a subject that has garnered significant attention in recent research [11-20]. Originating from the work of Cabral et al. in [15], this problem aims to minimize costs while ensuring that each source node can reach the sink node through a single route within limited transmission distances. Konak's extension [14] introduces a novel problem formulation incorporating a set covering constraints. This formulation forms the basis for a genetic algorithm featuring a specialized crossover/mutation operator, generating feasible paths for each commodity. The relay locations on these paths are then determined by solving the corresponding set covering problem. In [16], Xiao et al. propose a hybrid approach utilizing variable neighbourhood searches. The variable neighbourhood algorithm identifies routes for each source node, and an implicit enumeration algorithm determines optimal relay locations for a given set of routes. Li et al. [12] put forward an iterative meta-heuristic algorithm based on tabu search, addressing the NDPR problem in two steps. However, these studies primarily focus on transmission distance constraints and do not fully account for the operational dynamics of wireless sensor networks. In contrast, Bagaa et al. [19] and Li et al. [20] establish models for optimal relay node placement, specifically targeting the minimization of energy consumption in wireless signal transmission. Various works in the literature [21, 22] delve into relay node location problems considering signal coverage constraints. Naveen et al. [22] define signal coverage as a location within the communication range of a node eventually connected, possibly via multiple hops, to a sink node. Existing models studying signal coverage often rely on the ideal 0–1 disk channel model [23] and the latest Signal-to-Noise Ratio (SNR) model [17].

Nevertheless, these investigations predominantly concentrate on ascertaining whether all designated areas can be covered, with limited attention given to service quality and timely reliability [24]. In the realm of wireless networks, the paramount concern is whether user needs can be satisfied within specified timeframes. Hence, it becomes crucial to incorporate timely reliability considerations when modelling the location of base stations. Notably, Nigam et al. [11] and Naveen et al. [22] have delved into the optimal placement of relay nodes in wireless sensor networks, taking delay constraints into account. Their objective is to formulate a multi-hop wireless network that meets transmission path delays, ultimately minimizing the number of required relay base stations. However, these investigations uniformly employ the number of hops as the sole criterion for measuring network transmission delay and reliability. This approach overlooks the intricacies of the actual data transmission process and user requirements. Consequently, the results obtained from these studies may not directly offer actionable insights for communication operators.

One of the major challenges faced by current heuristic-based models in addressing the optimization of 5G base station locations is the accurate simulation of Line-of-Sight (LOS) propagation and the coverage of 5G signals in urban settings. Many existing location optimization models for cellular network planning were originally designed for 2G/3G/4G networks [25]. In urban areas, the penetration loss of 2G/3G/4G signals is considerably lower than that of 5G signals [26]. Consequently, the location optimization models developed for earlier generations of cellular networks did not need to account for the simulation of LOS propagation of signals.

Recent research endeavours have employed Geographic Information Systems (GIS) to facilitate the simulation of Line-of-Sight (LOS) propagation and coverage of 5G signals, offering support for optimization and enabling intuitive visual analyses in the planning of 5G networks. For instance, [27] utilized GIS alongside a computational geometry approach to optimize the locations of below-rooftop wall-mounted 5G base stations in urban outdoor areas, with GIS providing crucial support [28]. However, it is worth noting that the computational geometry approach may not be suitable for global optimization. Conversely, recent studies have introduced spatially implicit heuristic algorithm-based models to aid in 5G cellular network planning, particularly in specialized 5G application scenarios [29]. For example, the deployment of 5G base stations on unmanned aerial vehicles (UAVs) has been explored as an effective means to mitigate the penetration loss of 5G signals in urban areas. Consequently, these optimization models for UAV base station deployment do not involve the simulation of LOS propagation of 5G signals.

Exploring the optimization of base station locations for efficient coverage of the service demand area with the minimal deployment of 5G base stations, based on the spatial arrangement of buildings in urban outdoor settings, remains an area in need of further investigation. It is crucial to simulate LOS propagation and assess the service coverage of each base station to enhance decision-making in the planning of 5G networks [30, 31].

*4. I would recommend writting equations in new line (not inside the text) and numbering them properly (Section 2.1).*

Done, dear Collegue.

*5. Also, make sure that each parameter in every equation has been explained in the text.*

Dear Colleague, I carefully checked the text of Section 2 and made sure that all entered variables are described. In addition, for the convenience of the reader, I have introduced the Nomenclature at the end of the article.

*6. What are the limitations of the proposed approach?*

Dear Colleague, the article solves the problem of optimal placement of 5G base stations along a linear topology, taking into account the need to ensure seamless coverage and reliable communication. To achieve these conditions, a parametric space was introduced, described in Section 2.1. As a result, at the end of Section 2.1, extremal problem (1) was formulated with the following limitations:

1. For each pair $\left(p_{k},b_{i}\right)$:

- on the left: either there is a pair of $\left(p_{q},b_{j}\right)$ such that $x_{k}-x_{q}\leq S_{ij}$ and $x_{k}-x_{q}\leq S_{ji}$, or $x_{k}-x_{0}\leq S_{i0}$ and $x_{k}-x_{0}\leq S_{0i}$;

- on the right: either there is a pair of $\left(p_{g},b_{f}\right)$ such that $x_{g}-x_{k}\leq S_{if}$ and $x_{g}-x_{k}\leq S_{fi}$, or $x_{m+1}-x_{k}\leq S_{i(n+1)}$ and $x_{m+1}-x_{k}\leq S_{(n+1)i}$;

2. At each point from the set $Р$, no more than one base station from the set $В$ can be located;

3. The inequality $\sum_{i\in B_{\delta}} \bar{D}_{i}\leq D$ is satisfied, where $B_{\delta}$ is the set of deployed base stations from the set $В$, and $\bar{D}_{i}$ is the average data packet transmission delay determined for the base stations from the set $B_{\delta}$;

4. The total cost of base stations from the set $B_{\delta}$ does not exceed the value of $V$.

Note that the fulfilment of requirement 1 guarantees that each base station from the set $B_{\delta}$ will communicate with the gateways $\left\{b_{0},b_{n+1}\right\}$ either directly or through other stations of this set.

*7. If possible, provide comparisons to other available approaches.*

Thank you for this comment, dear Colleague. It is both difficult and simple for me to answer you. The difficulty is that I did not find a direct analogue for the mathematical apparatus presented in the article. In this regard, I have nothing to directly compare the results with. When I understood this problem, I realized that the material in Section 3 (Results) should be focused on justifying the adequacy of the mathematical apparatus proposed in Section 2. This result was achieved and commented on at the beginning of Section 4 (Discussion).

At the same time, I understand that scientific research implies a comparison of the results obtained to justify the effectiveness of the proposed approach. To highlight analogues in section 2.3 based on expression (6), two adaptations (*ROP, COP*) of the basic method (*BOP*) for solving the extremal problem (1) were proposed, which are characterized by less computational complexity, but are potentially less accurate than the basic method. This point is described in Section 4:

We will investigate the dependence of such qualitative indicators as the solution time $T$ [s], non-coverage $h$ [m] and solution accuracy $Acc$ [%], for the three options for calculating the term $r_{2}\left(W_{c}\right)$ from expression (6) described in Section 2.3, which determines the evaluation of the not-covering function $h\left(A\right)$ when solving the extremal problem (1) by the adapted branch-and-bounds method. For the definition $r_{2}\left(W_{c}\right)$, we proposed three variants of the formulation of the optimization problem (7):

- base (*BOP*, optimization problem (7) with constraints (8), (9): integer Linear Programming Problem),

- reduced (*ROP*, optimization problem (7) with constraint (8): integer Knapsack Problem),

- common (COP, optimization problem (7) with constraints (8), (9): ordinary Linear Programming Problem).

Among other initial parameters, note $l\left(\rho\right)=50$ [km], $\left|P\right|\in\left[5,30\right]$, $\left|B_{\pi}\right|\in\left[6,100\right]$, $V=600$ [c.u.] The rest of the parameters are identical to those entered in Section 3.

Thus, Section 4 presents a comparison of the base method with two analogues in {*T, h, Acc*}metric. The comparison results are visualized in Fig. 8-10. At the end of Section 4, corresponding conclusions are drawn:

From those presented in Figs. 8-10 results, it can be concluded that to estimate the term $r_{2}\left(W_{c}\right)$ from the expression (6) with a large dimension of the optimization problem (1), the statement $COP$ should be used. At the same time, we will play slightly lower in accuracy (see Fig. 10), but we will significantly (more than 6 times for large pairs $\left(\left|P\right|,\left|B_{\mu}\right|\right)$) win in the speed of obtaining a solution.

*8. Avoid using we/our throughout the paper.*

Dear Colleague, I agree that the original text of the article suffered from some literary and grammatical imperfections. At the same time, the English language is quite unclear (it’s a substitute to mention the British and American versions, modern and classical grammar, etc.) To improve the readability of the article, I hired a qualified translator and, as an independent expert, the Grammarly service.

**Response to the Reviewer #2 comments**

Dear Colleague,

I am sincerely grateful to you for the time you devoted to the article and for constructive criticism. I carefully revised the text of the article following your recommendations. I hope you will support the current version of the article.

With respect,

on behalf of the team of authors,

Viacheslav Kovtun.

*0. This paper highlights the need for an optimal planning model for the location of base stations; however, no novelty or innovation is observed compared to recent publications on georeferenced scenarios. It is suggested that the authors review planning works in georeferenced scenarios and expose the present research's added value.*

Thank you for this comment, dear Colleague. I completely agree with you. In the classical setting, the task of wireless base station localization is to locate them at permissible points of the target area to ensure maximum coverage. To solve such an optimization problem, computational geometry or heuristic approximate methods of global optimization (genetic algorithms, swarm algorithms, etc.) are usually used. The main reason for using these methods is that the said optimization problem has a large dimension. In such conditions, heuristic methods allow us to obtain a solution with an error, but within a certain fixed period. If I were solving such a problem, I would most likely use a combination of some exotic methods, for example, a bee algorithm, a water drop algorithm, etc. In the article under discussion, I do not solve the above-mentioned optimization problem.

The article under discussion solves a specific optimization problem formulated in the context of organizing a wireless network based on the 5G platform. The specificity is that the mentioned network is deployed along a linear segment, for example, a road, pipeline, or railway.

A special feature of the 5G platform is, among other things, the ability to implement several virtual network segments on the base station hardware using Network Slicing technology. In the model proposed in Section 2.1, it is assumed that the frequency resource of the base stations (the cost of which is known) is divided into two virtual segments, one of which is used to serve subscribers, and the second to ensure the network connectivity (for communication with other base stations). The first segment is characterized by higher throughput and a smaller coverage radius, and the second – vice versa. For the deployed network, parameters such as total cost and total delay are taken into account. Within the target linear segment, the coordinates of the points at which the base station can be located are known. The special same type of base stations (gateways) are located at the ends of the target segment.

The optimization problem is that it is necessary to place base stations on the target segment in such a way as to maximize the size of the telecommunications coverage of the segment while fulfilling the requirement for each station to communicate with gateways through a system of hosted stations, as well as fulfilling restrictions on the amount of end-to-end delay and the total cost of deployed stations. Thus, the wireless network structure obtained as a result of solving such an optimization problem will be reliable (due to constant communication between base stations and gateways) and efficient (due to maximizing coverage focused on serving subscribers). In addition, the gateway concept allows the integration of multiple line segments into a single network. This allows us to limit ourselves to the analysis of dozens of points of potential location of base stations in the process of searching for the optimal network configuration. In turn, this makes it possible to solve such an optimization problem using exact methods, among which the most suitable is the branch and bound method (subject to its adaptation to the above-formulated specifics of setting the optimization problem). In addition, the proposed mathematical apparatus takes into account that base stations (not gateways) can be of different types (this allows us to take into account the possibility of upgrading already functioning network architectures). Naturally, the last clarification further complicates the final optimization problem.

All of the above stated in the complex is a new research problem, as a result of which it is possible to design linearly organized network segments based on 5G base stations that meet the conditions of high reliability, high quality of service and rational use of funds. Separately, I note that in Section 2.4 a mathematical apparatus is proposed for calculating such technological parameters of the base stations used as communication range, end-to-end delay.

*1.- Introduction (Generalities of the problem - 15 references between 2024-2020)*

Dear Colleague, I fulfilled this suggestion within 11 sources ([1]-[11]).

*2.- Related Works (Specific works with punctual solutions, summary table of state of the art of other proposals against the current work - 15 references between 2024-2020)*

Dear Colleague, I diligently tried to follow this suggestion to the letter, but I could not. First of all, I have not found any direct analogues for solving the optimization problem, formulated in terms set out in p. 0. The approximate analogues I have found (sources [11]-[31]) are somewhat beyond the lower limit you defined. If, in your opinion, I have unfairly neglected relevant research, then I ask you to tell me doi. I also ask that you allow me to leave the analysis of analogues in the form of text and not a table. Still, textual analysis of analogues is more common in research publications than tables, which are more often found in reports. The result of my implementation of your suggestion is presented below.

The challenge of determining the optimal locations for 5G base stations can be conceptualized as a network design problem involving relays (NDPR), a subject that has garnered significant attention in recent research [11-20]. Originating from the work of Cabral et al. in [15], this problem aims to minimize costs while ensuring that each source node can reach the sink node through a single route within limited transmission distances. Konak's extension [14] introduces a novel problem formulation incorporating a set covering constraints. This formulation forms the basis for a genetic algorithm featuring a specialized crossover/mutation operator, generating feasible paths for each commodity. The relay locations on these paths are then determined by solving the corresponding set covering problem. In [16], Xiao et al. propose a hybrid approach utilizing variable neighbourhood searches. The variable neighbourhood algorithm identifies routes for each source node, and an implicit enumeration algorithm determines optimal relay locations for a given set of routes. Li et al. [12] put forward an iterative meta-heuristic algorithm based on tabu search, addressing the NDPR problem in two steps. However, these studies primarily focus on transmission distance constraints and do not fully account for the operational dynamics of wireless sensor networks. In contrast, Bagaa et al. [19] and Li et al. [20] establish models for optimal relay node placement, specifically targeting the minimization of energy consumption in wireless signal transmission. Various works in the literature [21, 22] delve into relay node location problems considering signal coverage constraints. Naveen et al. [22] define signal coverage as a location within the communication range of a node eventually connected, possibly via multiple hops, to a sink node. Existing models studying signal coverage often rely on the ideal 0–1 disk channel model [23] and the latest Signal-to-Noise Ratio (SNR) model [17].

Nevertheless, these investigations predominantly concentrate on ascertaining whether all designated areas can be covered, with limited attention given to service quality and timely reliability [24]. In the realm of wireless networks, the paramount concern is whether user needs can be satisfied within specified timeframes. Hence, it becomes crucial to incorporate timely reliability considerations when modelling the location of base stations. Notably, Nigam et al. [11] and Naveen et al. [22] have delved into the optimal placement of relay nodes in wireless sensor networks, taking delay constraints into account. Their objective is to formulate a multi-hop wireless network that meets transmission path delays, ultimately minimizing the number of required relay base stations. However, these investigations uniformly employ the number of hops as the sole criterion for measuring network transmission delay and reliability. This approach overlooks the intricacies of the actual data transmission process and user requirements. Consequently, the results obtained from these studies may not directly offer actionable insights for communication operators.

One of the major challenges faced by current heuristic-based models in addressing the optimization of 5G base station locations is the accurate simulation of Line-of-Sight (LOS) propagation and the coverage of 5G signals in urban settings. Many existing location optimization models for cellular network planning were originally designed for 2G/3G/4G networks [25]. In urban areas, the penetration loss of 2G/3G/4G signals is considerably lower than that of 5G signals [26]. Consequently, the location optimization models developed for earlier generations of cellular networks did not need to account for the simulation of LOS propagation of signals.

Recent research endeavours have employed Geographic Information Systems (GIS) to facilitate the simulation of Line-of-Sight (LOS) propagation and coverage of 5G signals, offering support for optimization and enabling intuitive visual analyses in the planning of 5G networks. For instance, [27] utilized GIS alongside a computational geometry approach to optimize the locations of below-rooftop wall-mounted 5G base stations in urban outdoor areas, with GIS providing crucial support [28]. However, it is worth noting that the computational geometry approach may not be suitable for global optimization. Conversely, recent studies have introduced spatially implicit heuristic algorithm-based models to aid in 5G cellular network planning, particularly in specialized 5G application scenarios [29]. For example, the deployment of 5G base stations on unmanned aerial vehicles (UAVs) has been explored as an effective means to mitigate the penetration loss of 5G signals in urban areas. Consequently, these optimization models for UAV base station deployment do not involve the simulation of LOS propagation of 5G signals.

Exploring the optimization of base station locations for efficient coverage of the service demand area with the minimal deployment of 5G base stations, based on the spatial arrangement of buildings in urban outdoor settings, remains an area in need of further investigation. It is crucial to simulate LOS propagation and assess the service coverage of each base station to enhance decision-making in the planning of 5G networks [30, 31].

*3.- Problem Formulation and Methodology (Table of variables of the mathematical model, mathematical model, pseudocode, methodology flowchart, Big Oh Notation of the algorithm - Forecasting),*

Dear Colleague, thank you for this comment. Indeed, Section 2 is full of many entered parameters, which are quite difficult to remember as you read the article. In response to this suggestion, I introduced a Nomenclature section before References. In addition, the material in Section 2.2 is supplemented with visualization of the Branching of a binary search tree and the Moving through the search tree processes (Fig. 1 and Fig. 2, respectively).

I support the concept of open science, dear Colleague. This is why I publish most of my articles in open journals: [**CV**](https://drive.google.com/file/d/19Fi34jL-kL-oEMcBDA5mNFLLagCfs_oG/view?usp=sharing). Likewise, I support open data (I like that PLoS makes data a mandatory part of the article). But science must earn funds. That is why the software (written in Python), which is built based on the mathematical apparatus presented in Section 2, is now at the stage of processing documents for intellectual property rights. In this regard, demonstration of the code is undesirable (however, I am ready to send you all the project files for personal use at any time you ask). Below I describe the main modules of the created software, a description of its structure and screen forms with the results of its functioning.

The structure of the created software package is shown in the figure below

The input to the software package is a JSON file containing Сonfiguration* parameters and Task input data**.

Before starting the placement task, it is necessary to prepare parameters for each base station (BS): communication radius between stations and BS coverage radius (Section 2.4). The parameters are calculated in the telecommunications range calculation block using the communication line energy potential equation and signal propagation models.

The base station placement problem is presented as a combinatorial model in extreme form. The algorithm for finding the optimal placement is of the branch and bound type. To find the optimal placement, a procedure for constructing a binary search tree is used. At each step, conditions are checked for the possibility of closing a tree node. The construction of a binary search tree is implemented recurrently.

As we move through the search tree, all received Optimal records are recorded (example in the figure below). The placement cost Cost, the end-to-end delay time Delay and the number of nodes in the tree (Node) where the record was obtained are also recorded. For each record, multiple station placements are recorded. The last record obtained is the optimal solution to the problem. The solution to the problem is written in the output JSON file:

* Configuration parameters are located under the configuration key:

– method: a method for solving the placement problem. The value bab is the branch and bound method or bf is the brute force method;

– estimation_method: a method for calculating the right under coverage estimate. The BOP value is a solution using the simplex method, the ROP value is a solution using an integer linear programming problem, or the COP value is a solution using the “Knapsack” problem (see Verification (7) in Section 2.3);

– placed_all_station: a special case of the placement problem, when there are no restrictions on cost and delay time, in such a setting the entire set of stations is placed. Accepts the values True or False;

– drawing: visualization of the resulting decision tree. Accepts the values True or False;

– last_optimal_noncoverage: the obtained optimal undercover on the last solution for the problem of finding a sequence of best solutions. Accepts any positive real values;

– relation_deviation: specified deviation in percentage from the obtained optimal solution for the problem of finding a sequence of best solutions.

** The JSON file contains the optimization Task input data:

– placement coordinates and gateway_placement coordinates;

– parameters of antennas of gateways and user_device subscriber devices;

– task restrictions on cost cost_limit and delay time delay_limit;

– parameters for calculating the range of telecommunication coverage: carrier frequency frequency, signal fading margin link_som and coverage_som;

– parameters for calculating delays: average packet size average_packet_size and intensity of incoming flow arrival_rate;

– sta – set of specified base stations.

Each element of the sta array is a JSON object and contains the following parameters:

– Ptr_link – the power of the antenna providing telecommunications communication between stations;

– Gtr_link – antenna gain that provides telecommunications between stations;

– Precv_link – sensitivity of the antenna providing telecommunications

communication between stations;

– L_link – signal loss in the cable and connectors of the transmitting path of the antenna, which provides telecommunications between stations;

– L_coverage – signal loss in the cable and connectors of the transmitting path of the antenna providing telecommunications coverage of the BS;

– Precv_coverage – sensitivity of the antenna providing telecommunications coverage of the BS;

– Grecv_coverage – a gain of the antenna providing telecommunications coverage of the BS;

– cost – the cost of the BS;

– throughput – BS throughput.

Finally, dear Colleague, I would like to note that a somewhat classical style of presentation of the material in Section 2 is generally accepted in the scientific school to which I have the honour of belonging. If the suggestion “methodology flowchart, Big Oh Notation...” is not fundamental for you, then I would ask you to leave the presentation style of Section 2 unchanged.

*4. - Analysis of Results (Metrics performed in Matlab in PDF format or directly from Overleaf)*

Dear Colleague, Section 3 presents the results of the software package I described above for optimizing the location of base stations on the linear segment. I would like to note that a fragment of one of the central streets of my hometown Vinnytsia (Ukraine) was taken as a linear segment. I do not link it to Google Maps because in conditions of active war such information could provoke an attack by the aggressor. The values of the input parameters required for the operation of the software complex, which I mentioned earlier in the description of Task input data, are determined at the beginning of Section 3. Next, using the material described in Section 2.4, the effective coverage radii for each station and the communication radii between stations and with gateways are calculated ( Fig. 3, Fig. 4). Next, the optimal placement is searched using the basic version of the adapted branch and bound method described in section 2.3. The calculation results are shown in Fig. 5. The following characteristics are used as metrics: the value of the non-coverage function h, the cost of implementation of the placement plan V, the delay D, the number of the vertex of the search tree N. Fig. 6 illustrates the evolution of base station placement plans (synchronous with the steps described in the qualitative metric in Fig. 5). Our adaptation of the branch and bound method (see expression (11)) provides the possibility of searching for an optimal placement that allows for under-covering part of the target linear segment (Δ, %). An example of the implementation of this feature is shown in Fig. 7.

As I mentioned earlier, based on expression (6) in Section 2.3, the article proposed three options for solving the optimization problem (7): estimation_method: a method for calculating the right under coverage estimate. The BOP value is a solution using the simplex method, the ROP value is a solution using an integer linear programming problem, or the COP value is a solution using the “Knapsack” problem. In Section 4, we compared the results of implementing these methods in the metric the solution time T, non-coverage h and solution accuracy Acc. The calculation results are presented in Fig. 8-10.

Finally, using the «method» attribute (method for solving the placement problem: - the value bab is the branch and bound method, - the value bf is the brute force method), implemented in the Configuration of our software package, I will compare the effectiveness of the authors’ adaptation of the branch and bound method with the brute-force method for the original optimization problem. Using the «drawing» attribute (visualization of the resulting decision tree. Accepts the values True or False) we get visual data for comparison.

The tree obtained as a result of solving the original problem using the proposed adaptation of the branch and bound method:

The tree obtained as a result of solving the original problem using the brute force method:

Thus, to obtain the same exact solution to the original optimization problem, the brute force method turned out to be significantly slower. This trend will only increase as the parametric space of the original optimization problem increases.

I want to emphasize once again that I chose the exact branch and bound method to solve the optimization problem formulated in the article, and not any heuristic method, because the formulation of the problem provides for dozens, not hundreds of solution points. For such cases, exact methods not only generally work faster than heuristic ones, but also allow one to obtain an exact solution. Finally, I will mention once again that the article proposes an adaptation of the branch and bound method, which allows us to obtain an optimal plan for the placement of base stations with a controlled amount of under coverage of the target linear segment.

*5.- Conclusions (Direct relation between the objective stated in the abstract vs. the metrics found)*

Done, dear Collegue.

*6.-References 2024-2020 (All documents with DOI from ScienceDirect, MDPI, Wiley, PLOS, Taylor & Francis, Springer, Hindawi, IEEE Xplore [transactions, magazines, journals]).*

Done, dear Colleague (subject to the exceptions highlighted by me during my answer to suggestion 2).
